# Measurement of the intracellular lifetime of water to estimate myocardial cell size is not feasible in humans using clinical contrast agent doses at 1.5T

**DOI:** 10.1186/1532-429X-18-S1-P237

**Published:** 2016-01-27

**Authors:** Magnus Lundin, Peder Sörensson, Peter Kellman, Andreas Sigfridsson, Martin Ugander

**Affiliations:** 1 Department of Clinical Physiology, Karolinska Institutet, Stockholm, Sweden; 2 Department of Molecular Medicine and Surgery, Karolinska Institutet, Stockholm, Sweden; 3 National Heart, Lung, and Blood Institute, National Institutes of Health, Bethesda, MD USA

## Background

Measurement of the myocardial extracellular volume fraction (ECV) assumes a dynamic equilibrium with rapid exchange of an extracellular contrast agent between myocardium and blood. This equilibrium would be manifested by a linear relationship between 1/T1 (R1) of myocardium and 1/T1* (R1*) of blood. This relationship has been shown to be non-linear for high R1 values in mice, and this has been used to calculate the intracellular lifetime of water and estimate myocardial cell size. We sought to determine whether the relationship between R1 of myocardium and R1* of blood is linear or non-linear in patients scanned under clinical conditions.

## Methods

Consecutive patients referred for clinical cardiovascular magnetic resonance (CMR) evaluation of suspected heart disease were prospectively enrolled. CMR was undertaken at 1.5T (Siemens Aera) using a modified Look-Locker inversion recovery (MOLLI) sequence before and approximately 3, 8 and 16 minutes after an intravenous bolus of a gadolinium-based extracellular contrast agent (Dotarem, gadoteric acid, 0.2 mmol/kg). Patients were included if the CMR findings were normal and there were no severe artefacts. Patients underwent T1- and T1*-mapping with MOLLI. A region of interest was placed in the left ventricular blood pool (T1*) and midmurally in the myocardium (T1) of a midventricular short-axis slice at all time points.

## Results

In the study population (n=16, age 52 ± 18 years, 63% male), there was a linear relationship between R1 of myocardium and R1* of blood (R1 myocardium = 0.441 x R1* blood + 0.726, R2=0.979, *p*<0.001, see figure). Non-linear regression (R1 myocardium = 0.338 x R1* blood^1.13 + 0.835, R2=0.981, *p*<0.001) did not differ from linear regression (*p*=0.93 for t-test of absolute residuals). Similar results were obtained when using R1 instead of R1* of blood. Mean ± SD T1 of the myocardium was 981 ± 27 ms pre-contrast, 307 ± 32 ms 3 minutes post-contrast, 419 ± 27 ms 8 minutes post-contrast, and 475 ± 32 ms 16 minutes post-contrast.

## Conclusions

There is a linear relationship between the relaxation rate of the myocardium (R1) and the relaxation rate in the blood pool (R1*), for patients with normal MRI findings. A non-linear model did not improve the fit of the data. This is true as early as three minutes after double dose intravenous contrast bolus administration. Clinical measurement of the intracellular lifetime of water will require a dedicated imaging protocol, likely with a contrast agent dose exceeding current clinical routine.

**Figure 1:**
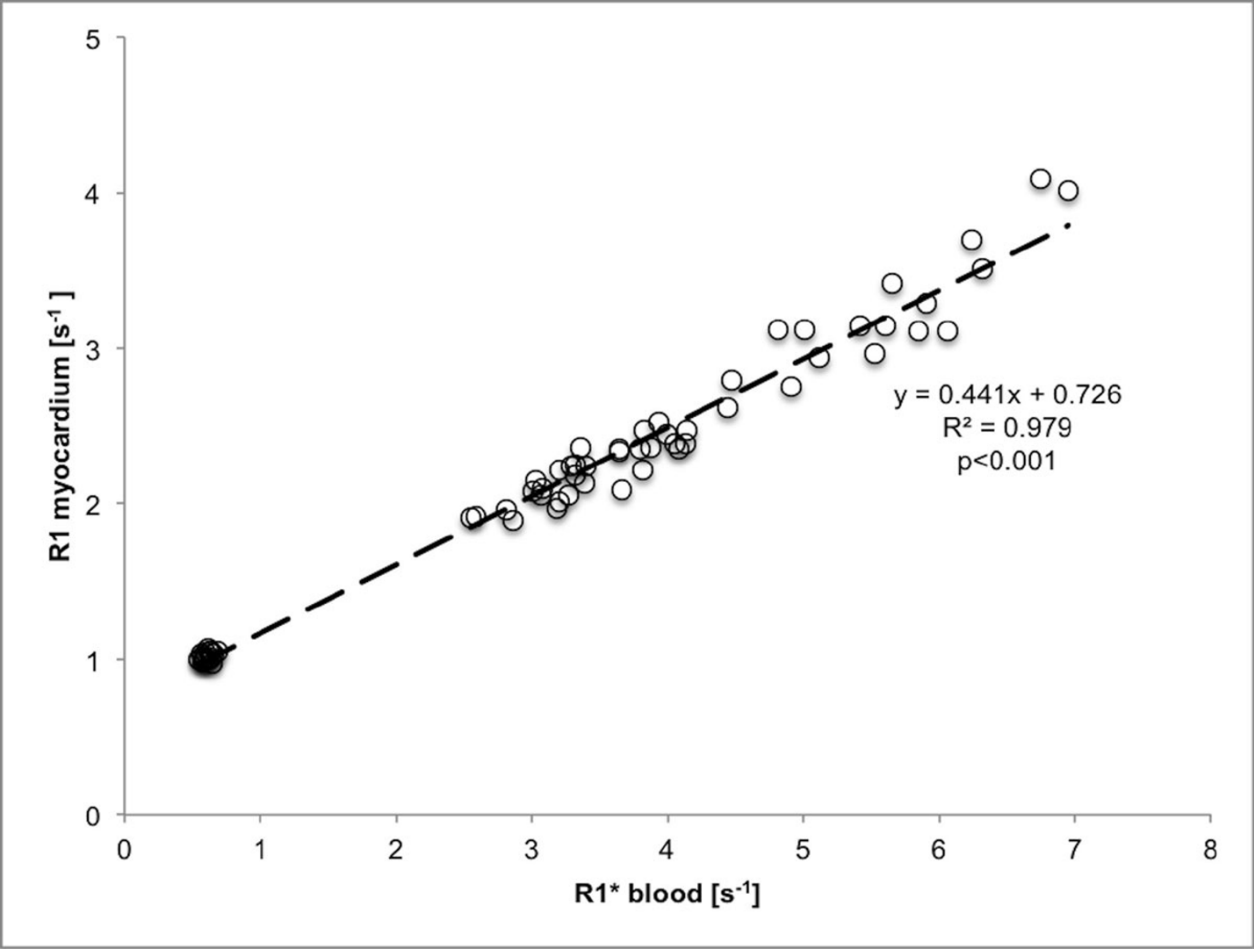
R1 of myocardium plotted against R1* of the blood pool.

